# Human Milk Biology: Illuminating the Path toward the Next Generation of Infant Formula

**DOI:** 10.1016/j.advnut.2026.100723

**Published:** 2026-08-21

**Authors:** Andrew A Bremer

**Affiliations:** Office of Nutrition Research, National Institutes of Health, Bethesda, MD, United States

Human milk remains the biological gold standard for infant nutrition because it is far more than a source of nutrients—it is a dynamic biological system that coordinates immune development, microbial colonization, metabolism, neurodevelopment, and tissue maturation [1,2]. Advances in systems biology, analytical chemistry, glycobiology, multiomics, and computational science have transformed our understanding of milk from a relatively static nutritional fluid into an adaptive biological network that continuously responds to maternal, infant, and environmental influences [[1], [2], [3], [4]]. In their accompanying article, “Human milk as a model for next-generation infant formula: opportunities and challenges”, Biondi Ryan et al. [5] synthesize these advances and propose a roadmap for translating discoveries in milk biology into the development of future infant formulas.

Their central premise is both timely and compelling: advancing infant formula requires more than reproducing the nutrient composition of human milk—it requires understanding and, where possible, reproducing its biological functions. Human milk contains thousands of interacting constituents, including proteins, lipids, human milk oligosaccharides (HMOs), extracellular vesicles, immune cells, hormones, enzymes, microorganisms, and signaling molecules that function collectively within an integrated biological matrix [[1], [2], [3], [4]]. Infant development is therefore shaped not by individual components acting in isolation but by coordinated biological interactions between milk, the infant, and the surrounding environment.

This system perspective to human milk and its implication on next-generation infant formula development is extremely important. Nutrition science has traditionally relied on reductionist approaches that identify individual nutrients responsible for specific physiological effects. Although this paradigm has generated foundational discoveries, it cannot fully explain the remarkable biological complexity of human milk. Increasing evidence instead supports viewing milk as an adaptive ecosystem whose function emerges from interactions among maternal physiology, milk composition, infant biology, the developing microbiome, and environmental influences [[1], [2], [3], [4]]. This concept formed the foundation of the NIH-supported Breastmilk Ecology: Genesis of Infant Nutrition project [[2], [3], [4]] and similarly underpins the forthcoming NIH–Food and Drug Administration Nutritional Ecology of Early Development project, which extends this ecological framework to infant feeding and early-life nutrition.

Substantial progress has already been made in infant formula development. The incorporation of long-chain PUFAs, bovine milk fat globule membrane, lactoferrin, osteopontin, probiotics, and increasingly complex HMOs reflects the successful translation of discoveries in human milk biology into nutritional innovation [[6], [7], [8]]. Yet, meaningful biological differences between human milk and infant formula remain and likely contribute to persistent differences in immune maturation, gastrointestinal development, neurodevelopment, and long-term health.

Emerging technologies provide unprecedented opportunities to narrow this biological gap. Precision fermentation enables the production of recombinant human milk proteins and structurally identical HMOs, whereas advances in mammary epithelial organoids, tissue engineering, and bioactive ingredient isolation are creating increasingly sophisticated strategies for reproducing biological function rather than simply nutrient composition. These innovations represent an important conceptual shift—from designing formulas that resemble human milk to developing formulas that emulate its biology.

Achieving this goal, however, requires a deeper understanding of milk itself. Milk composition exhibits remarkable temporal and interindividual variability driven by maternal genetics, nutrition, metabolic health, stage of lactation, circadian rhythms, environmental exposures, and infant demand [[1], [2], [3], [4]]. Defining what constitutes “normal” human milk—and distinguishing adaptive biological variation from random variability—remains one of the field’s central scientific challenges.

Human milk research is therefore entering a new era. After decades devoted primarily to cataloging milk constituents, investigators are increasingly positioned to define biological function. Rather than asking whether a molecule is present, researchers can now examine how coordinated molecular networks regulate immune maturation, microbial ecology, intestinal development, metabolism, neurodevelopment, and lifelong health [[1], [2], [3], [4]]. Integrating multiomics technologies with longitudinal maternal–infant phenotyping, advanced computational biology, and artificial intelligence promises to identify functional biological networks and molecular signatures that were previously inaccessible.

The implications of these advances in human milk research extend well beyond infant formula as well. Human milk represents perhaps the most sophisticated example of precision nutrition in nature, delivering an evolving combination of nutrients and bioactive signals precisely matched to the developmental needs of the infant. Deciphering this biology promises advances not only in neonatal nutrition but also in immunology, microbiome science, developmental programming, metabolism, neuroscience, and systems biology [[1], [2], [3], [4]].

Importantly, advancing infant formula should complement—not replace—efforts to support breastfeeding. Millions of infants worldwide depend partially or entirely on formula because of maternal illness, prematurity, insufficient milk production, adoption, or social and structural barriers to breastfeeding. Improving infant formula therefore represents an important public health priority, with human milk continuing to serve as the biological reference standard for infant nutrition. Continued investment in evidence-based lactation support and breastfeeding promotion remains equally essential [9].

The article by Biondi Ryan et al. [5] also underscores the importance of modern regulatory science. As infant formulas become increasingly biologically sophisticated, evaluation frameworks must evolve beyond nutrient adequacy and growth to incorporate functional outcomes such as immune development, microbiome maturation, neurodevelopment, metabolic programming, and long-term health. Achieving this goal will require validated biomarkers, standardized methodologies, and sustained collaboration among academia, industry, and regulatory agencies [10].

The future of infant nutrition will not be defined by identifying the next bioactive ingredient to add to infant formula, but by understanding the biological systems that make human milk uniquely capable of supporting infant development. Human milk should serve not only as the biological reference standard for infant feeding but also as the prototype model for system nutrition. By embracing this conceptual framework, investigators will establish the scientific foundation for the next generation of infant formulas while advancing precision nutrition during one of the most critical periods of the human life course. The perspective by Biondi Ryan et al. [5] represents an important step toward that future.

## Author contributions

The sole author was responsible for all aspects of this manuscript.

## Declaration of Generative AI and AI-assisted technologies in the writing process

During the preparation of this work, the author used ChatGPT Gov in order to edit the text for clarity and concision. After using this tool/service, the author reviewed and edited the content as needed and takes full responsibility for the content of the publication.

## Funding

The author reported no funding received for this study.

## Conflict of interest

The contents of this article represent the author’s views and do not constitute an official position of the National Institutes of Health or the United States Government. The author is an Editor of Advances in Nutrition and played no role in the Journal’s evaluation of the manuscript.
